# Supplementary material for: MetaRibo-Seq measures translation in microbiomes
Source: Nat Commun. 2020 Jun 29;11:3268. doi: 10.1038/s41467-020-17081-z (PMC7324362; doi:10.1038/s41467-020-17081-z)
Supplement: Supplementary file 10 — Supplementary Data 7 [file 41467_2020_17081_MOESM10_ESM.zip › File2/Confidence_High_Taxonomy/416265_out.krona.html]

Javascript must be enabled to view this page.

members
magnitude
magnitudeUnassigned
count
unassigned
taxon
rank

416265\_out

9

superkingdom
9
2

544448
phylum
9

31969
class
9

186329
9
order

9
species
2053494

SRS014613\_contig\_number\_contig-100\_340.129269SRS017821\_contig\_number\_33473SRS023914\_contig\_number\_contig-100\_2082.145792SRS054905\_contig\_number\_20968SRS055017\_contig\_number\_37119SRS143991\_contig\_number\_10411SRS144537\_contig\_number\_contig-100\_1818.246287SRS147271\_contig\_number\_35883SRS147346\_contig\_number\_56494
